# A Pre-pandemic Baseline: Assessing Gaps in Sexually Transmitted Infection Knowledge Among Healthcare Providers at the Obstetrics and Gynecology Department of a Saudi Tertiary Care Hospital

**DOI:** 10.7759/cureus.101634

**Published:** 2026-01-15

**Authors:** Lamyaa Majed, Kamal Adwan, Rasha Majed, Somaya Adwan

**Affiliations:** 1 Obstetrics and Gynecology, Saudi Commission for Health Specialties, Jeddah, SAU; 2 Medicine and Surgery, Batterjee Medical College, Jeddah, SAU; 3 General Surgery, United Doctors Hospital, Jeddah, SAU; 4 Biostatistics and AI, Universiti Malaya, Kuala Lumpur, MYS

**Keywords:** a cross-sectional study, healthcare providers, knowledge assessment, molecular vaccines and diagnostics, obstetrics & gynecology, pre-pandemic baseline, sexual transmitted diseases

## Abstract

Background: Assessing the competency of healthcare providers in managing sexually transmitted infections (STIs) is critical for reproductive health. This 2019 study established a pre-pandemic baseline by evaluating the knowledge, diagnostic, and management practices of healthcare providers in the Department of Obstetrics and Gynecology (OB/GYN) at King Abdulaziz University Hospital (KAUH) in Jeddah, Saudi Arabia.

Methods: A descriptive cross-sectional study was conducted among 136 of 156 eligible physicians (87.2% response rate). A validated, self-administered questionnaire assessed knowledge across seven STIs: syphilis, gonorrhea, chlamydia, chancroid, herpes simplex virus (HSV), human papillomavirus (HPV), and human immunodeficiency virus (HIV). Data were analyzed using descriptive statistics and chi-square (χ²) goodness-of-fit tests against a 50% chance-level benchmark to determine if knowledge scores differed significantly from random guessing.

Results: Significant knowledge gaps were identified. While STI recognition varied (93.4% for syphilis vs. 58.1% for chancroid), critical deficits existed in applying modern guidelines. Only 47.1% recognized the often-asymptomatic nature of STIs. Low proportions of participants identified first-line interventions: 34.6% correctly cited ceftriaxone for gonorrhea, 32.4% knew polymerase chain reaction (PCR) was optimal for HSV, and merely 16.9% identified nucleic acid amplification tests (NAATs) for chlamydia. Awareness of pre-exposure prophylaxis (PrEP) was low (24.3%), despite high condom-use acknowledgment (90.4%).

Conclusion: This study establishes a crucial pre-pandemic baseline, identifying critical knowledge gaps in guideline-based STI management among OB/GYN professionals, including recommended diagnostics and treatments. These findings highlight an urgent need for targeted, continuous medical education to improve clinical competency and patient safety, offering a benchmark for measuring future educational interventions and pandemic-related disruptions on clinical practice.

## Introduction

Sexually transmitted infections (STIs) represent a monumental global public health challenge, with the World Health Organization (WHO) estimating over one million new infections acquired daily, leading to profound consequences for sexual, reproductive, and neonatal health, including infertility, cervical cancer, and adverse pregnancy outcomes [1,2]. An effective defense against this burden hinges directly on a proficient clinical workforce. The competency of healthcare providers in STI management, encompassing accurate syndrome recognition, knowledge of modern diagnostic tools, application of evidence-based treatment regimens, and effective patient counseling, is therefore a critical determinant of national STI control efforts [3].

Recognizing this, the Saudi Arabian health authorities have formally acknowledged the importance of STI competency. The Saudi Commission for Health Specialties (SCFHS) has integrated "Sexually transmitted infection, prevention, screening, diagnosis, and management" into its national curricula for relevant specialties, including obstetrics and gynecology (OB/GYN) and preventive medicine [4,5]. However, the inclusion of a topic in a curriculum does not guarantee that healthcare providers can competently apply that knowledge when diagnosing and treating patients. A significant and concerning gap appears to exist between this policy-level mandate and the translational effectiveness of medical education and training.

Evidence from diverse geographical and clinical settings has consistently revealed significant deficiencies in physician knowledge regarding STIs, establishing this as a widespread global challenge. International bodies like the WHO and the Centers for Disease Control and Prevention (CDC) have established comprehensive guidelines for STI management, underscoring the critical need for a knowledgeable clinical workforce [6-8].

Nevertheless, a substantive gap exists between theoretical curriculum and applied clinical competency, as evidenced by studies across multiple regions. Research from Pakistan, conducted over 10 years apart, has demonstrated persistent limitations in general practitioners' understanding, highlighting not merely a local educational shortfall but a universal need for continuous education [9,10]. This pattern is firmly echoed within the Middle East and North Africa region. A systematic review highlighted a significant lack of focus on the competencies of healthcare practitioners themselves in the Arabian Peninsula, indicating a critical blind spot in the regional research agenda [11]. Further supporting this, family physicians in Kuwait were found to have notable knowledge gaps and negative attitudes toward human immunodeficiency virus (HIV)/AIDS [12].

This challenge is not new to Saudi Arabia; knowledge gaps among physicians have been documented for decades. As early as 1995, primary healthcare physicians demonstrated significant unmet educational needs and concerning knowledge levels regarding AIDS [13,14], indicating long-standing systemic issues in training and knowledge retention. These foundational gaps are compounded by persistent problematic attitudes among healthcare professionals, such as the stigmatization of persons living with HIV/AIDS, which has been identified as a barrier in the literature for over 10 years [15-19].

This indicates that the challenges are not merely academic but are deeply embedded in the clinical culture and attitudes, which educational interventions have yet to fully overcome. Furthermore, a recent 2022 study of primary healthcare physicians in Jeddah revealed that knowledge of the syndromic management of STIs remains variable, with particularly low competency in managing syndromes like genital ulcers and vaginal discharge in pregnant women [20]. This pattern, spanning from historical knowledge deficiencies to enduring stigmatizing attitudes and current gaps in standardized management protocols, underscores a deep-seated and multifaceted challenge within the Saudi healthcare system.

Saudi Arabia presents a distinct epidemiological profile where increasing case notifications of STIs [16,19] coincide with persistent gaps in public knowledge and awareness. A 2023 national study underscored significant variations and misconceptions about sexual health and STIs among women in the Kingdom [21], aligning with earlier, localized findings [21,22]. This shortfall in public awareness amplifies the responsibility of healthcare professionals to deliver accurate screening, counseling, and care.

The OB/GYN department serves as a critical frontline in STI management. OB/GYN providers operate at the nexus of routine women’s healthcare, prenatal care, and reproductive tract infection management and control. Their frequent contact with sexually active women affords essential opportunities for screening, early diagnosis, and preventive education. Consequently, gaps in their core STI knowledge directly compromise this strategic function, increasing the likelihood of missed diagnoses, inadequate treatment, and further transmission.

Therefore, deficiencies in their foundational STI knowledge, including disease recognition, diagnostic pathways, and treatment protocols, can have disproportionately severe consequences. These gaps directly increase the risk of missed screenings during prenatal care, failure to prevent mother-to-child transmission, and inadequate management leading to infertility, ectopic pregnancy, and other long-term reproductive tract sequelae. Despite this imperative, a critical and specific gap persists in the Saudi literature. While studies have focused on epidemiological reporting [16,19], the knowledge of the general public [17,21,22], or the knowledge and attitudes of physicians toward specific infections like HIV [13-15,18], a comprehensive, in-depth evaluation of STI-specific knowledge among OB/GYN healthcare providers is conspicuously absent. No study has yet systematically assessed this key specialist group across the full spectrum of STI knowledge, from pathogenesis and symptomatology to modern diagnostics, first-line treatments, and advanced prevention strategies. This represents a severe blind spot, as the competence of these core practitioners is fundamental to achieving national sexual and reproductive health goals.

Therefore, to address this pivotal gap, this research aimed to establish a detailed pre-pandemic baseline by performing a holistic assessment of STI knowledge among healthcare providers in the OB/GYN department of a major tertiary care hospital in Jeddah, Saudi Arabia. By benchmarking current knowledge levels against both international standards [6-8] and the competencies expected by national bodies [4,5], this study seeks to determine the scale of the deficit, identify specific areas where educational translation has failed, and provide empirical evidence to guide a crucial revision of training and implementation strategies to ensure that policy-level commitments translate into effective clinical practice. The remainder of this paper is structured as follows: the Methods section details the study design and analytical approach, the Results section presents the findings of the knowledge assessment, the Discussion section interprets these findings in the context of existing literature and policy, and the Conclusion summarizes key findings and recommendations.

## Materials and methods

Study design

A descriptive, cross-sectional study was conducted between February 1 and August 31, 2019, to assess knowledge of STIs among healthcare providers in the Department of Obstetrics and Gynecology at King Abdulaziz University Hospital (KAUH) in Jeddah, Saudi Arabia. This design was selected as it provides a "snapshot" of the knowledge levels at a specific point in time, establishing a crucial pre-pandemic baseline. The single-center setting, while providing a detailed departmental benchmark, may limit the generalizability of the findings to other institutions. Ethical approval was obtained from the Unit of Biomedical Ethics Research Committee at the King Abdulaziz University Faculty of Medicine (Reference No: 266-19). The study protocol adhered to the principles of the Declaration of Helsinki. Written informed consent was secured from all participants, and strict confidentiality was maintained throughout the study.

Study setting and population

The study was conducted at KAUH and targeted a census of all 156 physicians working in the OB/GYN department. A total of 136 physicians participated, yielding a high response rate of 87.2%. This high response rate minimizes the potential for non-response bias and provides a highly representative picture of the knowledge levels within the entire departmental population. While this census approach ensures representativeness for the overall cohort, comparisons between smaller subgroups may have limited statistical power.

Sample size calculation and justification

**Figure 1:**
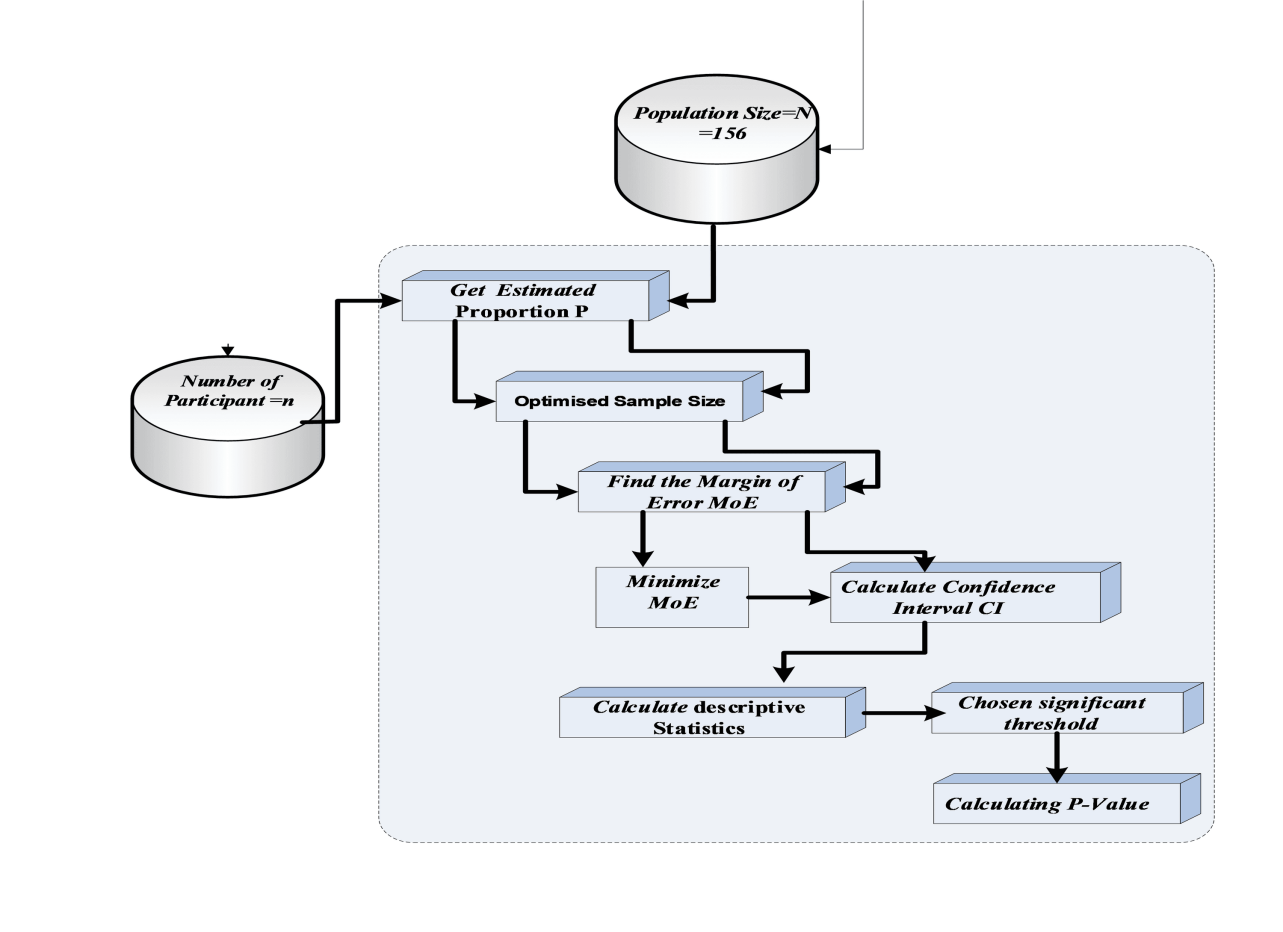
Workflow for optimized sample size calculation and statistical analysis This flowchart illustrates the sequential, stepwise statistical methodology employed to determine the study cohort and analyze the collected data. The process begins by defining the target population size and an estimated proportion (p) for key variables. These parameters were used to calculate an optimized sample size, ensuring sufficient statistical power. The subsequent steps focus on minimizing the MoE and calculating a precise CI for the findings. The final stages of the workflow outline the core analytical procedures: calculating descriptive statistics to summarize the data, selecting a predefined significance threshold (α, typically 0.05), and deriving p-values through hypothesis testing. This visual guide provides a clear roadmap of the analytical approach, underscoring the methodological rigor applied to ensure the statistical validity of the study's inferences. MoE, margin of error

Based on a target population (N) of 156 physicians, a minimum sample size was calculated a priori using a standard formula for cross-sectional studies (MATLAB 2021). The calculation assumed a 95% confidence level (Z=1.96), a conservative response distribution of 50% to maximize variability, and a margin of error of 5%. This indicated a minimum required sample size of 110. The study successfully recruited 136 participants, exceeding this target and enhancing the representativeness and precision of the findings for estimating overall knowledge prevalence. It is important to note that this calculation was designed for descriptive prevalence estimation; the study was not specifically powered for inferential statistical comparisons between all demographic or professional subgroups. Therefore, such subgroup analyses presented later should be considered exploratory. The stepwise statistical workflow for sample size determination, margin of error minimization, and inference planning is detailed in Figure 1.

Data collection tool and development

Data were collected using a structured, self-administered questionnaire developed specifically for this study through a multistage process. The initial draft was based on an extensive review of existing literature and internationally recognized guidelines from the WHO and the U.S. CDC [1,2,4-6], ensuring content validity and relevance. The questionnaire was then reviewed by a panel of five senior consultants in obstetrics/gynecology, infectious diseases, and public health to assess its face and content validity, and their feedback was used to refine the questions. A pilot test was conducted on a small group of 10 physicians (not included in the final sample) to identify ambiguities and assess functionality. The final questionnaire is presented in Appendix 1. It consisted of four distinct sections: Section A (Socio-demographic and Professional Characteristics); Section B (Validation and Prior Education); Section C (STI-Specific Knowledge Assessment); and Section D (Knowledge of Preventive Measures). Data collection employed a dual-mode approach, using both paper-based questionnaires during a dedicated seminar and an identical digital version via Google Forms to maximize participation.

Analytical framework: phase-based knowledge assessment

**Figure 2:**
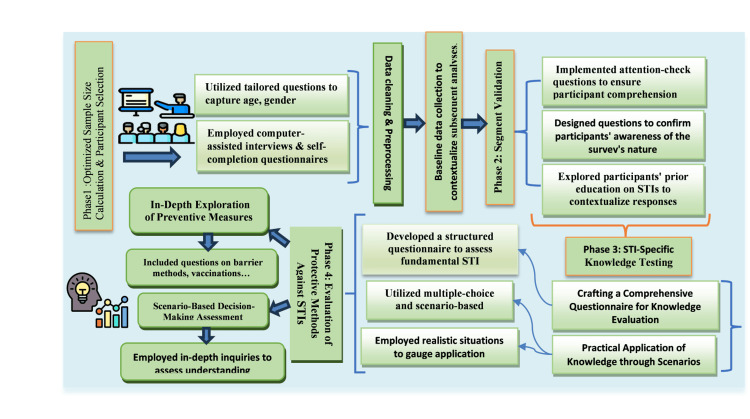
Schematic diagram of the four-phase analytical framework This visual schematic outlines the structured, sequential methodology employed to analyze questionnaire data. The framework comprises four distinct phases that transform raw responses into stratified, clinically actionable findings. Phase 1, Demographic Profile and Sample Representation, established the cohort's baseline characteristics. Phase 2, Data Integrity Verification and Educational Baseline, authenticated responses and documented prior training to ensure a validated dataset. Phase 3, Clinical Knowledge Competency Assessment, formed the core evaluation, testing knowledge across seven STIs against current guidelines. Phase 4, Preventive Strategy Proficiency Evaluation, assessed understanding of prevention methods. The diagram illustrates how each phase builds upon the verified output of the previous one, ensuring that the final analysis is grounded in contextually rich and methodologically sound data. This framework provided the logical roadmap for the results presented in this study. STI, sexually transmitted infection

Following the statistical planning outlined in Figure 1, data analysis employed a structured, four-phase sequential framework illustrated in Figure 2, which systematically transformed raw questionnaire responses into stratified, clinically meaningful insights. The process began with Phase 1, Demographic Profile and Sample Representation. In this phase, data from Section A of the questionnaire were analyzed to establish the cohort's baseline characteristics, ensuring sample representativeness and providing essential context for subsequent analysis. Phase 2, Data Integrity Verification and Educational Baseline, followed. This phase analyzed Section B to authenticate response quality through built-in attention-validation mechanisms and to document participants' prior formal training in STI management, thereby creating a validated dataset and establishing an educational baseline to contextualize knowledge scores. The core evaluation occurred in Phase 3, Clinical Knowledge Competency Assessment. Utilizing Section C, this phase performed a multidimensional evaluation across seven STIs. Knowledge was assessed across eight standardized domains per infection, with correctness determined against contemporary U.S. CDC guidelines [1,2,6], evaluating both theoretical understanding and practical application. The final stage, Phase 4, Preventive Strategy Proficiency Evaluation, examined Section D to gauge comprehension of STI prevention. This involved a systematic assessment across the prevention spectrum, from fundamental barrier methods and vaccination awareness to advanced biomedical interventions such as pre-exposure prophylaxis (PrEP). This phased architecture ensured each analytical layer was built upon a verified and properly contextualized foundation.

Data management and statistical analysis

Data from the paper questionnaires were manually entered, consolidated into a single master file, cleaned, and coded for analysis. Statistical analysis was performed using SPSS Statistics version 28 and MATLAB R2021a. The analysis proceeded as follows: descriptive statistics, including frequencies and percentages, were used to summarize demographic characteristics and responses to each knowledge item.

For inferential analysis, chi-square (χ²) goodness-of-fit tests were employed. The expected proportion of 50% was chosen as a conservative benchmark representing a state of no knowledge (pure chance), allowing us to determine if collective performance on an item was systematically better or worse than random guessing, using a p-value threshold of <0.05 for statistical significance. The results of this analysis emphasize the clinical magnitude and precision of the estimates, reporting the observed proportions of correct responses alongside their 95% CI and chi-square statistics.

To synthesize these findings into a strategic overview, the data were subsequently visualized using MATLAB R2021a (The MathWorks, Inc., Natick, MA, USA). The resulting "Knowledge Landscape" graphically represents the variation in knowledge proficiency across the seven STIs, visually highlighting key areas of strength and critical gaps for intervention. This comprehensive, sequential methodological approach, from descriptive and inferential statistics to integrative visualization, ensured the collection and analysis of robust, valid, and reliable data, forming a solid foundation for the findings and conclusions of this study.

## Results

The findings of this study are presented according to the four-phase analytical framework established in the methodology, progressing from the demographic landscape of the respondents to a granular assessment of their clinical knowledge and preventive strategies.

Phase 1: demographic profile and sample representation

**Figure 3:**
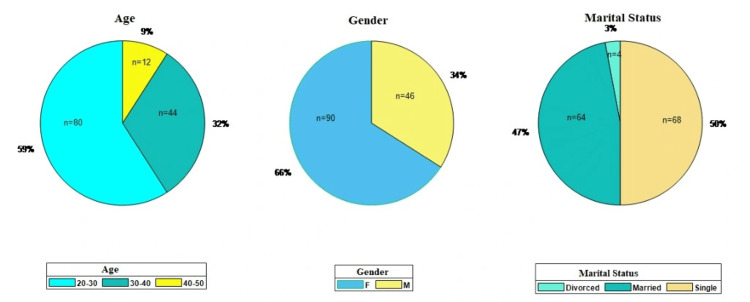
Demographic distribution of survey participants This pie chart visualization presents the demographic composition of the healthcare provider sample (n=136). It displays the percentage breakdown across three key variables: age group (highlighting the predominant 20-30-year cohort), gender (showing the majority as female, "F"), and marital status (categorized as single, married, or divorced). The chart allows for the immediate assessment of the study population's structure, which is crucial for contextualizing the subsequent knowledge and behavioral analyses. The data confirm that the sample is predominantly young, female, and single, reflecting a specific demographic profile within the OB/GYN department setting. OB/GYN, obstetrics and gynecology

The survey achieved a high participation rate, with 136 out of 156 eligible physicians completing the questionnaire, yielding a robust response rate of 87.2% (136/156). The demographic profile of these respondents, illustrated in Figure 3, depicts a cohort predominantly composed of early-career clinicians; a total of 80 participants (58.8%) were aged 20-30 years, and 44 participants (32.4%) were aged 30-40 years, comprising primarily residents and junior specialists. The gender distribution reflected the specialty's composition in the region, with 66% of participants being female. The sample was nearly evenly divided between single (68 participants, 50.0%) and married (64 participants, 47.1%) participants, providing a representative cross-section of the department's clinical staff.

Phase 2: response validation and baseline education

This phase served the dual purpose of authenticating data integrity and establishing a crucial baseline of participants' prior educational exposure, both essential for contextualizing the subsequent knowledge assessment. To ensure response reliability, the questionnaire incorporated a validation segment featuring non-STI pathologies, specifically cutaneous leishmaniasis and vulvovaginal candidiasis. The finding that over 95% of participants correctly identified these conditions as non-sexually transmitted demonstrated a high level of genuine comprehension and engagement, thereby validating the dataset for further analysis.

Concurrently, an analysis of self-reported prior education revealed a distinct hierarchy in training focus that powerfully foreshadowed the knowledge gaps identified later. As shown in Table 1, prior education was highest for HIV (86.4%, n=51/59) and syphilis (79.7%, n=47/59), followed closely by gonorrhea and chlamydia (78.0% each). In contrast, human papillomavirus (HPV) (76.3%) and herpes simplex virus (HSV) (74.6%) received moderately less emphasis. In contrast, chancroid was the least covered, with only 64.4% (n=38/59) of providers reporting any formal training. This systematic educational disparity provides a compelling explanation for the severe knowledge deficits observed in Phase 3, particularly for chancroid, highlighting a specific weakness in current training paradigms that prioritize certain infections over others of clinical importance.

**Table 1:** Knowledge of selected STIs among healthcare providers: frequency of correct responses and chi-square test results This table presents the number (n), percentage (%), and 95% CI of correct responses for each knowledge domain across seven STIs. For core knowledge items (n=136), chi-square (χ²) goodness-of-fit tests were conducted to test if the observed proportion of correct answers significantly differed from an expected chance level of 50%. The corresponding 95% CIs provide the precision of each estimate; intervals entirely above 50% indicate performance significantly better than chance, while intervals entirely below 50% indicate systematic misconception. Prior education rows (n=59) are presented descriptively without statistical testing or CI. STI, sexually transmitted infection; PCR, polymerase chain reaction; HPV, human papillomavirus; DNA, deoxyribonucleic acid; VDRL, venereal disease research laboratory; RPR, rapid plasma reagin; NAATs, nucleic acid amplification tests; HIV, human immunodeficiency virus; ELISA, enzyme-linked immunosorbent assay; HAART, highly active antiretroviral therapy

HSV knowledge	Correct answers (n)	%	Chi-square (χ²)	95% CI	
Is a STI	95	69.85	21.44		(61.6, 77.1)
Yes, received education about it	44	74.6	-		(61.6, 84.5)
It is not curable	33	24.3	36.03		(17.5, 32.3)
It is reportable	27	19.9	49.44		(13.7, 27.6)
It has no available vaccine	89	65.44	12.97		(56.9, 73.2)
Mostly presented by primary genital ulcer	29	21.3	44.74		(15.0, 29.1)
Condoms do not protect against it completely	41	30.1	21.44		(22.7, 38.6)
Best be tested by PCR	44	32.4	16.94		(24.7, 40.9)
Acyclovir is the basic treatment	91	66.9	15.56		(58.5, 74.5)
HPV knowledge					
Is a STI	116	85.29	67.76		(78.2, 90.5)
Yes, received education about it	45	76.27	-		(63.4, 86.0)
It is not curable	36	26.47	30.12		(19.3, 34.7)
It is reportable	51	37.5	8.50		(28.2, 47.6)
It has available vaccine	109	80.15	49.44		(72.5, 86.3)
Mostly presented by warts	88	64.7	11.76		(56.2, 72.5)
Condoms do not protect against it completely	41	30.1	21.44		(22.7, 38.6)
Best be tested by DNA testing	26	19.1	51.88		(13.0, 26.8)
Podophyllin is one of the treatment options	51	37.5	8.50		(29.3, 46.3)
11-12 years is the recommended age for HPV vaccine	23	16.9	59.56		(11.1, 24.4)
Syphilis knowledge					
Is a STI	127	93.4	102.38	(87.7, 96.8)	
Yes, received education about it	47	76.27	-	(67.2, 88.5)	
It is curable	110	80.9	51.88	(73.3, 86.9)	
It is reportable	73	53.7	0.74	(45.1, 62.1)	
It has no available vaccine	106	77.9	42.47	(70.1, 84.4)	
Chancre present with primary syphilis	49	36.03	10.62	(28.1, 44.6)	
Screened by VDRL/RPR	107	78.7	44.74	(70.9, 85.0)	
Best be tested by treponemal tests	69	50.7	0.03	(42.1, 59.3)	
Benzathine penicillin G is the basic treatment	85	62.5	8.50	(53.9, 70.5)	
Chlamydia knowledge					
Is a STI	114	83.8	62.24	(76.5, 89.4)	
Yes, received education about it	46	78	-	(65.3, 87.3)	
It is curable	118	86.8	73.53	(79.8, 91.7)	
It is reportable	38	27.9	26.47	(20.7, 36.3)	
It has no available vaccine	110	80.9	51.88	(73.3, 86.9)	
Mostly presented with mucopurulent discharge	43	31.6	18.38	(24.0, 40.2)	
Best be tested by NAATs	23	16.9	59.56	(11.1, 24.4)	
Azithromycin or doxycycline is the basic treatment	64	47.1	0.47	(38.6, 55.6)	
Gonorrhea knowledge					
Is an STI	123	90.4	88.97		(84.1, 94.7)
Yes, received education about it	46	78	-		(65.3, 87.3)
It is curable	120	88.2	79.53		(81.5, 93.0)
It is reportable	55	40.4	4.97		(32.2, 49.2)
It has no available vaccine	105	77.2	40.26		(69.3, 83.8)
Urethritis in men is the most common presentation	35	25.7	32.03		(18.7, 34.0)
Best be tested by swab culture	52	38.2	7.53		(30.2, 46.9)
Ceftriaxone is the basic treatment	47	34.6	12.97		(26.8, 43.2)
HIV knowledge					
Is an STI	110	80.9	51.88		(73.3, 86.9)
Yes, received education about it	51	86.4	-		(74.8, 93.7)
It is not curable	93	68.83	18.38		(60.0, 75.9)
It is reportable	115	84.6	64.97		(77.4, 90.0)
It has no available vaccine	114	83.8	62.24		(76.5, 89.4)
Presented with opportunistic infections	88	64.7	11.76		(56.2, 72.5)
Screened by ELISA	79	58.1	3.56		(49.4, 66.4)
Best be tested by Western blot assay	79	58.1	3.56		(47.2, 64.3)
HAART is the best treatment	96	70.6	23.06		(62.3, 77.8)
Chancroid knowledge					
Is an STI	79	58.1	3.56	(49.4, 66.4)	
Yes, received education about it	38	64.4	-	(51.0, 76.2)	
It is curable	68	50	0.00	(41.4, 58.6)	
It is reportable	26	19.1	51.88	(13.0, 26.8)	
It has no available vaccine	98	72.1	26.47	(63.8, 79.2)	
Presented with painful genital ulcers with inguinal lymphadenopathy	39	28.7	24.74	(21.3, 37.2)	
Best be tested by tissue culture	23	16.9	59.56	(11.1, 24.4)	
Ceftriaxone or azithromycin as a treatment option	38	27.9	26.47	(20.7, 36.3)	
Note: Prior education rows (n=59) are descriptive only and not included in statistical tests. 95% CI for these rows is based on n=59					

Phase 3: STI-specific clinical knowledge assessment

This phase provided a detailed evaluation of clinical competencies across the seven STIs, revealing profound and specific deficits in essential diagnosis and management areas, as compiled in Table 1. The assessment began by identifying a fundamental misconception of STI pathology; only 64 participants (47.1%) recognized the predominantly asymptomatic nature of common STIs. 

For HSV, while 95 participants (69.9%) recognized its sexually transmitted nature, 80.1% were unaware of its reportability to public health authorities. Clinical identification was particularly poor, with only 29 participants (21.3%) accurately associating HSV with primary genital ulcer formation, and a significant portion (32.35%) misattributed the classic presentation of a painful ulcer with lymphadenopathy to this virus. Knowledge of modern diagnostics was severely limited; only 44 participants (32.4%) identified polymerase chain reaction (PCR) as the most suitable diagnostic method for HSV, though 91 (66.9%) correctly recognized acyclovir as a therapeutic agent.

Regarding HPV, high awareness (85.3%, n=116) was contradicted by a significant misconception, as 67 participants (49.3%) erroneously believed the infection is curable. Although most participants were aware of the HPV vaccine (109/136, 80.15%), far fewer knew the recommended adolescent vaccination age (16.9%, n=23). Diagnostic knowledge was critically low, with only 26 participants (19.1%) aware of DNA testing, while 88 participants (64.7%) incorrectly associated warts with high-risk oncogenic strains. In terms of treatment, only 51 participants (37.5%) recognized podophyllin as a topical treatment option. Furthermore, only 41 participants (30.1%) knew that condoms do not provide complete protection against HPV and HSV. Syphilis was the most recognized STI (93.4%, n=127), and most providers were aware of its curability (80.9%, n=110). However, knowledge of confirmatory treponemal tests (fluorescent treponemal antibody-absorption test (FTA-ABS) and treponema pallidum particle agglutination assay (TP-PA)) was substantially lower (50.7%, n=69), and only 85 participants (62.5%) identified benzathine penicillin G as the first-line treatment.

Knowledge of chlamydia revealed a contrast between general awareness and specific clinical understanding. While most participants were aware that it is an STI (83.8%, n=114) and curable (86.8%, n=118), detailed clinical and diagnostic knowledge was lacking. Correct identification of mucopurulent discharge as a key symptom was low (31.6%, n=43). Diagnostic knowledge was particularly poor: only 23 participants (16.9%) identified nucleic acid amplification tests (NAATs) as the optimal test, whereas 43 (31.6%) incorrectly selected swab culture. Less than half of the participants (47.1%) were aware of the first-line treatment options.

Notably, knowledge of gonorrhea management was low; despite 90.4% (n=123) recognition and known curability (88.24%), only 47 participants (34.6%) identified ceftriaxone as the recommended first-line treatment (χ²=8.85, p=0.003), and only 38.2% knew culture-based techniques are the most accurate diagnostic method, while a quarter of participants (25.0%) admitted a complete lack of knowledge on the subject. Awareness of symptoms like urethritis in men was low (25.0%).

HIV knowledge was relatively more robust. Most providers recognized it (80.88%, n=110) and knew its incurable nature (68.38%, n=93). A strong majority understood its mandatory reportability (84.6%, n=115), with 96 participants (70.6%) identifying highly active antiretroviral therapy (HAART) as the optimal treatment, and 79 participants (58.1%) knowing the two-tiered diagnostic algorithm of enzyme-linked immunosorbent assay (ELISA) screening and Western blot confirmation tests. In a complex scenario, 102 participants (75.0%) opposed breastfeeding for HIV-positive mothers, while 25 participants (18.4%) supported it, with hesitancy often stemming from considerations of viral load and regional context.

**Figure 4:**
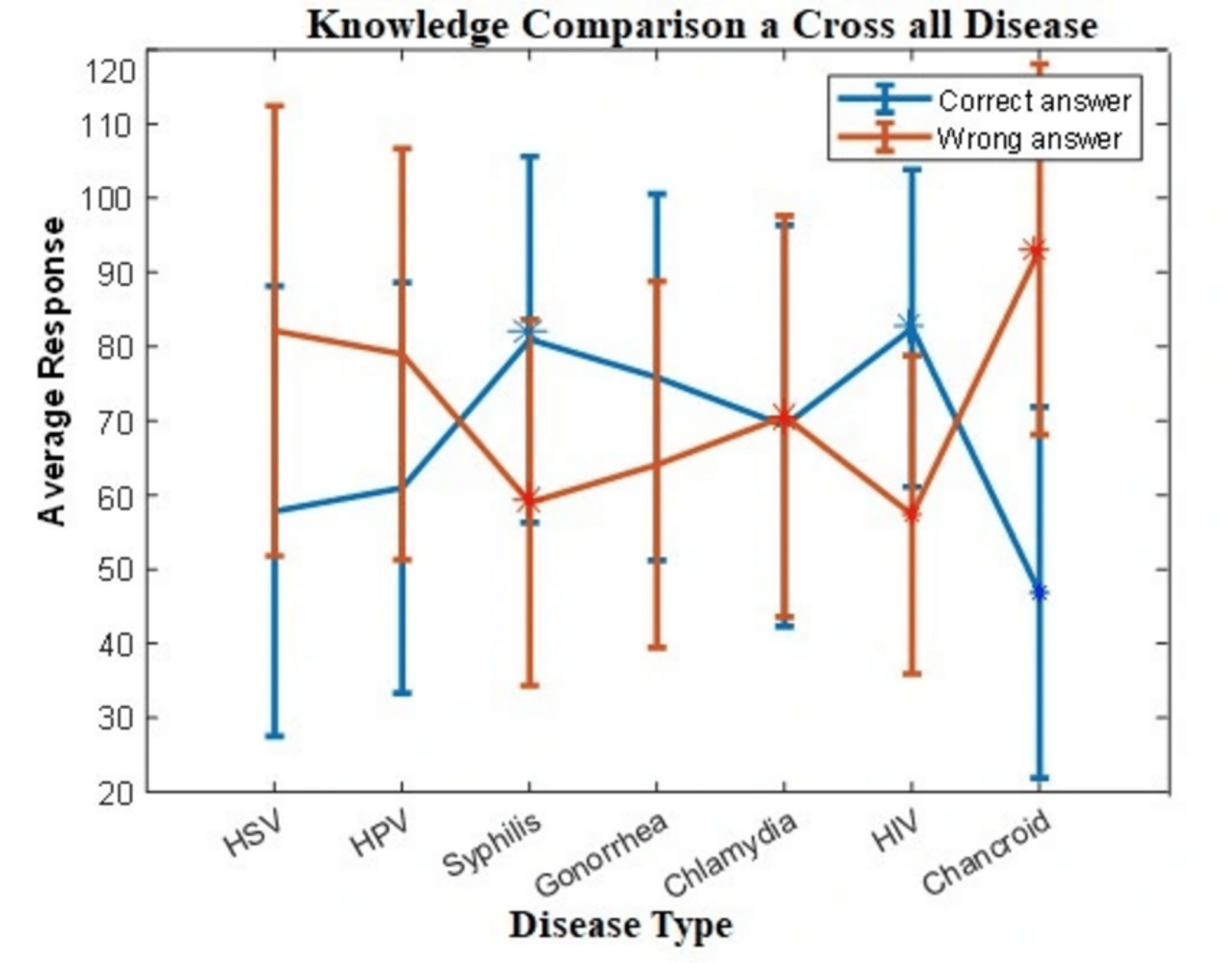
Knowledge landscape: illustrating peaks of knowledge and pits of misconception, maximum and minimum points for correct and incorrect answers across seven STIs This visualization was created by plotting the average percentage of correct answers for each of the seven assessed STIs, calculated from all relevant knowledge items per infection in the questionnaire. The line traces the variation in overall knowledge proficiency across diseases. Blue circles mark the STIs with the highest aggregate correct scores (peaks of knowledge), while red circles mark those with the lowest scores (valleys of misconception). This graphical synthesis transforms the detailed results from Table 1 into an actionable overview of departmental strengths and priority gaps. HSV, herpes simplex virus; HPV, human papillomavirus; STI, sexually transmitted infection; HIV, human immunodeficiency virus

Finally, chancroid demonstrated the most severe and comprehensive knowledge gaps, having the lowest recognition rate (58.1%, n=79), and only 39 participants (28.7%) were familiar with its pathognomonic clinical feature of a painful genital ulcer with regional lymphadenopathy. Diagnostic knowledge was critically low, with only 23 participants (16.9%) familiar with tissue culture techniques. Therapeutic knowledge was virtually absent, with nearly half of all participants (47.8%) unable to identify any appropriate treatment. The collective results detailed in Table 1 are synthesized in the "Knowledge Landscape" visualization (Figure 4), which graphically highlights the peaks and pits of knowledge across the infections. Overall, chi-square tests comparing composite knowledge scores found no statistically significant difference in performance across the seven STIs, as indicated by the p-values in Table 2.

**Table 2:** Overall knowledge comparison across STI categories and preventive methods This table provides a synthesized statistical summary of knowledge proficiency across the different STI areas and preventive methods. For each category, the table presents the aggregate performance: the total correct answers given, the total possible correct answers, the calculated overall percentage correct, and the corresponding 95% CI. The 95% CI indicates the precision of the overall estimate for each category. The 50% benchmark (representing chance-level performance) is used as a reference; a CI lying entirely above 50% indicates collective knowledge systematically better than chance (a relative strength), while an interval entirely below 50% indicates a systematic deficit. This analysis helps prioritize educational interventions by highlighting which broad topic areas represent the most critical foundational weaknesses based on both the magnitude and precision of the knowledge scores. HSV, herpes simplex virus; HPV, human papillomavirus; STI, sexually transmitted infection; HIV, human immunodeficiency virus

Knowledge area	Total correct/total possible	Overall % correct	Chi-square (χ²)	95% CI
HSV knowledge	503 / 1224	41.1%	30.24	(38.3, 43.9)
HPV knowledge	641 / 1360	47.1%	1.42	(44.3, 50.0)
Syphilis knowledge	733 / 1224	59.9%	111.01	(57.1, 62.6)
Gonorrhea knowledge	547 / 952	57.5%	44.94	(54.2, 60.7)
Chlamydia knowledge	510 / 952	53.6%	9.68	(50.3, 56.9)
HIV knowledge	749 / 1224	61.2%	127.88	(58.4, 63.9)
Chancroid knowledge	371 / 952	39.0%	48.26	(35.9, 42.2)
Protective methods of STIs	828 / 1360	60.9%	121.74	(58.2, 63.5)

Phase 4: knowledge of preventive strategies

The assessment of preventive knowledge, quantified in Table 3, revealed a clear gradient of proficiency, delineating strong awareness of fundamental public health measures from significant deficits in knowledge of advanced biomedical interventions. Participants demonstrated near-universal command of traditional behavioral strategies, with 126 participants (92.6%) correctly identifying reducing sexual partner numbers as an effective measure, and 123 participants (90.4%) affirming the role of condoms in blocking pathogen transmission. However, this high proficiency contrasted sharply with an understanding of more contemporary methods. While 91 providers (66.9%) recognized vaccination as a preventive strategy, comprehension of pharmacologic interventions was markedly deficient. Only 33 participants (24.3%) identified prophylactic antiviral drugs (PrEP) as an effective biomedical strategy for HIV prevention. A significant proportion of participants (32.4%, n=44) incorrectly endorsed male circumcision as a general protective measure against STIs. Furthermore, only 45 participants (33.1%) correctly identified cervical diaphragms as nonprotective against most STI pathogens, a finding that was statistically significant (χ²=6.83, p=0.009). In a consistent finding, an overwhelming majority of providers (131 participants, 96.3%) endorsed population-level screening and awareness programs as fundamental to STI control.

**Table 3:** Knowledge of STI protective methods among healthcare providers: frequency of correct responses and the corresponding chi-square test results This table quantifies participants' understanding of various strategies for preventing STIs. For each listed method, the table shows the number (n), percentage (%), and 95% CI of the 136 participants who correctly identified its protective or nonprotective efficacy. The 95% CIs convey the precision of each estimate. Responses were evaluated using chi-square goodness-of-fit tests against a 50% chance-level benchmark; CI entirely above or below 50% reflect collective knowledge or systematic misconception, respectively, for each preventive measure. STIs, sexually transmitted infections

Protective methods of STIs	Correct answers (n)	%	Chi-square (χ²)	95% CI
Preexposure vaccination is protective	91	66.9	15.56	(58.5, 74.5)
Abstinence is protective	74	54.4	1.06	(45.8, 62.8)
Avoiding multiple partners is protective	126	92.6	98.94	(86.8, 96.3)
Condoms are protective	123	90.4	88.97	(84.1, 94.7)
Cervical diaphragm is NOT protective	45	33.1	15.56	(25.4, 41.7)
Topical microbicides or spermicides are NOT protective	82	60.3	5.76	(51.7, 68.4)
Hysterectomy is NOT protective	111	81.6	54.38	(74.1, 87.5)
Male circumcision is protective	44	32.4	16.94	(24.7, 40.9)
Emergency contraception is NOT protective	104	76.5	38.12	(68.5, 83.1)
Prophylaxis antiviral drugs is protective	33	24.3	36.03	(17.5, 32.3)

## Discussion

This study establishes the first documented pre-pandemic baseline of STI knowledge among OB/GYN healthcare providers at KAUH in Saudi Arabia. The findings reveal clinically significant deficiencies that extend beyond basic awareness to encompass critical gaps in diagnostic and therapeutic competencies. Chi-square goodness-of-fit tests confirmed that the observed distribution of correct answers for most knowledge items significantly deviated from a 50% chance-level expectation (p < 0.05). The 95% CI for these proportions, reported in Tables 1-3, provides a range of plausible values for the true knowledge levels and further substantiates that these deviations are not only statistically significant but also clinically meaningful in magnitude. This rigorous quantification underscores that the identified patterns represent systematic strengths and deficiencies within the cohort, rather than random variation.

A foundational gap in clinical understanding was the low recognition of the predominantly asymptomatic nature of STIs, acknowledged by only 47.1% (95% CI: 38.6-55.6) of providers, a proportion whose CI lies entirely below the 50% chance threshold. This misconception directly undermines syndromic management approaches endorsed by the WHO and national guidelines, suggesting that a substantial proportion of infected patients may remain undiagnosed, thereby perpetuating silent transmission chains.

Equally concerning are the deficits in knowledge of first-line diagnostics and treatments, which raise substantial concerns for patient safety and antimicrobial resistance (AMR). Only a minority of providers correctly identified NAATs as the optimal diagnostic for chlamydia (16.9% (n=23), CI: 11.1-24.4) and ceftriaxone as the first-line treatment for gonorrhea (34.6% (n=47), CI: 26.8-43.2). The CI for both estimates is narrow and far below the 50% benchmark, indicating precise and profound deficits that reveal a critical misalignment with current international standards [6-8]. These gaps represent failures in clinical delivery that can contribute directly to diagnostic inaccuracy, therapeutic inefficacy, and the escalation of AMR.

The disconnect between established guidelines and applied knowledge points to a systemic educational issue. While the SCFHS has formally integrated STI management into the national curriculum [4,5], which signifies a crucial policy-level commitment, the pervasive knowledge gaps uncovered here, consistent with other regional findings [13,14,20], indicate that the current framework is insufficient for ensuring clinical competency. The challenge is twofold: the curriculum may lack necessary depth, and its delivery fails to guarantee translation into practice. To bridge this gap, we propose a fundamental shift toward a competency-based educational model. This would mandate a revised SCFHS curriculum emphasizing mastery of modern diagnostics and treatments, reinforced by longitudinal assessment through clinical simulations and standardized practical exams to enforce proficiency from training through continuous practice.

The "Knowledge Landscape" visualization (Figure 4) provides a powerful spatial representation of these disparities by plotting the average correct scores for each STI. This visual synthesis, derived directly from the aggregate questionnaire data, transforms complex statistical findings into an actionable educational roadmap. In the visualization, peaks represent areas of relative knowledge strength, exemplified by syphilis recognition (93.4%, χ²=102.38, n=127) and HIV treatment knowledge (70.6% identifying HAART, χ²=23.06, n=96). Conversely, the valleys identify critical gaps requiring immediate intervention, most notably the profound deficiency in chancroid management (58.1% recognition, χ²=3.56, n=79) and the substantial gaps in modern diagnostics (16.9% for NAATs in chlamydia; 32.4% for PCR in HSV). This graphically intuitive representation, grounded in the validated assessment data, not only confirms the heterogeneity of knowledge across STIs but also provides a strategic blueprint for prioritizing educational interventions.

Synthesized analysis (Table 3) further contextualizes these gaps. While knowledge in areas like HIV (61.2% correct) and syphilis (59.9% correct) was systematically above chance, performance for HPV (47.1% correct, p=0.233) was statistically indistinguishable from random guessing. The finding that nearly half of the participants considered HPV infection "curable" highlights a key educational gap. While clinical management of HPV-related lesions (e.g., warts, dysplasia) is possible and spontaneous viral clearance is common, current guidelines emphasize that no therapy eradicates the underlying HPV infection. This distinction is crucial for accurate patient counseling regarding transmission, persistence, and the preventive role of vaccination. This indicates a profound, nonsystematic misunderstanding lacking even a baseline consensus. Demographic analysis revealed no significant association between knowledge scores and professional rank or experience, suggesting a department-wide, systemic educational challenge requiring comprehensive rather than targeted interventions. These findings demonstrate that knowledge gaps previously documented in primary care settings [15-20] are equally severe among OB/GYN specialists, the providers entrusted with managing the complex reproductive sequelae of these infections.

The identified foundational knowledge gaps carry particular weight within the OB/GYN context. For instance, failure to recognize asymptomatic infections or to apply first-line diagnostics like NAATs for chlamydia can lead to missed screening opportunities during routine prenatal visits. This, in turn, increases the risk of untreated infections progressing to pelvic inflammatory disease, tubal factor infertility, ectopic pregnancy, and adverse pregnancy outcomes. Therefore, remedying these basic knowledge deficits is a critical prerequisite for effective specialty-specific care and the prevention of long-term reproductive morbidity.

The primary strength of this study is its methodical design, a phased analytical framework combined with robust statistical validation (including chi-square benchmarking and CI), which establishes a crucial pre-pandemic (2019) baseline of STI knowledge. Conducted as a census within a major tertiary center, this detailed assessment provides a precisely defined departmental benchmark. Although the single-center design may limit broad generalizability and the analysis was not powered for formal inferential comparisons between small demographic subgroups, this focused approach creates an essential reference point. It will enable future measurement of the impact of educational interventions within similar settings and help distinguish long-term knowledge gaps from pandemic-related service disruptions.

Collectively, this research makes three pivotal contributions. First, it shifts the focus from general awareness to specific, statistically validated clinical deficits, such as the critically low recognition of first-line diagnostics for chlamydia and treatment for gonorrhea, that directly compromise patient safety and antimicrobial stewardship. Second, it provides an indispensable pre-pandemic baseline against which the impact of subsequent curricular reforms and systemic disruptions can be evaluated. Third, it demonstrates a practical model for competency assessment by deploying a structured analytical framework to transform survey data into an actionable "knowledge landscape." This model offers healthcare authorities and educational bodies an evidence-based roadmap to champion targeted continuous professional development (CPD), underscoring the urgent need to translate curricular standards into measurable clinical proficiency at the frontline of care.

Building directly on this established baseline, we recommend a future follow-up assessment using the same methodology. Such a replication would allow health authorities to quantify post-pandemic recovery in service delivery and, crucially, to isolate persistent educational deficits from temporary disruptions.

## Conclusions

This study, employing a structured four-phase analytical framework, systematically quantified critical knowledge deficiencies in STI management among OB/GYN healthcare providers at KAUH. The findings extend beyond simple awareness to reveal statistically significant, domain-specific deficits in modern diagnostics and first-line treatments that directly compromise patient safety, antimicrobial stewardship, and the effectiveness of national STI control programs. The evidence, validated through chi-square analysis and synthesized in a clear "knowledge landscape," establishes an imperative for immediate educational reform.

To address these gaps, we recommend a dual-strategy intervention integrated into OB/GYN departmental structures: first, the inclusion of updated STI competency modules in mandatory grand rounds and simulation training; and second, the implementation of clinic-based decision aids and audit tools reinforced by practical assessment. Healthcare authorities and educational bodies must leverage this statistically grounded baseline to strengthen guidelines, allocate resources efficiently, and champion a systematic shift from curricular standards to measurable clinical proficiency. Ultimately, this study provides both a crucial pre-pandemic benchmark and a validated methodological model to guide policy, ensuring that frontline OB/GYN care is aligned with contemporary public health priorities.

## Appendices

Structured knowledge assessment questionnaire

Introduction for Participants

Dear colleague, you are invited to participate in a research study titled “Assessing the knowledge about sexually transmitted infections and their diagnostic methods, prevention, and behavior towards STI-positive patients among healthcare providers.” This survey is conducted by researchers at King Abdulaziz University and aims to evaluate current understanding and clinical practices related to STIs. Your anonymous and confidential participation is voluntary and highly valuable. By proceeding with this questionnaire, you provide your informed consent to participate. Please answer all questions to the best of your ability.

Section A: Socio-Demographic and Professional Characteristics

This section collects foundational information to contextualize your responses. Individual demographic and professional variables may be associated with differences in knowledge and are essential for analyzing the data across relevant participant subgroups.

- Age

- Gender

- Ethnicity

- Religious affiliation

- Marital status

- Confirmation of healthcare provider status

- Professional profile: Including medical specialty, clinical rank (e.g., consultant, specialist, resident), years of clinical experience, primary workplace sector (governmental/private), and facility type.

- International work experience: To understand exposure to diverse clinical guidelines and practices.

Section B: Response Validation and Assessment of Prior Education

This section serves two methodological purposes. First, it includes internal validation items (e.g., identifying non-STI conditions) to ensure attentive and genuine participation. Second, it documents your baseline level of formal education on each STI, which is crucial for interpreting knowledge scores.

Attention validation: Participants were asked to identify which of several listed conditions are primarily sexually transmitted; this list included common non-STI pathologies (e.g., bacterial vaginosis and leishmaniasis).

Prior training: Participants self-reported whether they had received any formal education or specific training on each of the seven core STIs covered in this study.

Section C: Comprehensive STI-Specific Knowledge Assessment

This is the core assessment module. It evaluates in-depth knowledge across eight critical clinical and public health domains for seven key STIs: syphilis, gonorrhea, chlamydia, chancroid, herpes simplex virus (HSV), human papillomavirus (HPV), and human immunodeficiency virus (HIV). Correct answers were benchmarked against current U.S. Centers for Disease Control and Prevention (CDC) guidelines. For each of the seven STIs, knowledge was assessed on:

Transmissibility:* *Recognition of the infection as sexually transmissible.

Curability: Knowledge of whether the infection is curable with appropriate treatment.

Reportability: Awareness of its status as a reportable disease to public health authorities.

Vaccination: Knowledge of vaccine availability.

Clinical presentation: Matching the infection to its cardinal symptom or sign.

Diagnostic modality: Matching the infection to its gold-standard or primary confirmatory test.

First-line treatment: Matching the infection to its recommended first-line pharmacotherapy.

Obstetric complications: Identifying potential adverse outcomes associated with the infection in pregnant patients. (The complete item-by-item breakdown for the matching questions in Domains 5-8 is presented in Table 1 of the article.)

Section D: Knowledge of Preventive Measures and Clinical Management Attitudes

This section transitions from factual knowledge to applied understanding and professional attitudes. It evaluates comprehension of prevention strategies and explores decision-making in clinical and ethical scenarios relevant to STI management.

Prevention strategies: Participants rated the efficacy of various preventive measures (e.g., vaccination, condoms, abstinence, and PrEP (pre-exposure prophylaxis)/PEP (post-exposure prophylaxis)).

Condom efficacy: Specific knowledge on the protection afforded by condoms against HSV and HPV.

Clinical screening practices: Self-reported triggers for initiating STI screening in patients.

Patient and partner management: Recommended steps following a patient's STI diagnosis, including partner notification.

Ethical and clinical scenarios: Attitudes regarding partner disclosure rights, breastfeeding advice for women with HIV, and protocols for communicating an STI diagnosis to a patient.

Public health perspective: Perceived importance of population-level screening and public awareness campaigns. (The exhaustive list of preventive methods evaluated is provided in Table 2 of the article.)

Conclusion for Participants

Thank you for your time and valuable contribution to this research. Your responses will help inform educational strategies and clinical practices to improve STI management.
